# 2,3-Dimethyl-1*H*-imidazol-3-ium benzene­sulfonate–1,2-dimethyl-1*H*-imidazole co-crystal

**DOI:** 10.1107/S2414314620006896

**Published:** 2020-05-27

**Authors:** Noah Cyr, Matthias Zeller, Patrick C. Hillesheim, Arsalan Mirjafari

**Affiliations:** a Department of Chemistry and Physics, Florida Gulf Coast University, 10501 FGCU Blvd. South, Fort Myers, FL, 33965, USA; b Purdue University, Department of Chemistry, 560 Oval Drive, West Lafayette, Indiana USA, 47907, USA; c Ave Maria University, Department of Chemistry and Physics, 5050 Ave Maria Blvd, Ave Maria FL, 34142, USA; Howard University, USA

**Keywords:** crystal structure, tosyl­ate, imidazolium, ionic crystal

## Abstract

In the title co-crystal, C_5_H_9_N_2_^+^·C_6_H_5_O_3_S^−^·C_5_H_8_N_2_, the two 1,2-di­methyl­imidazole rings exist as partially protonated moieties in the asymmetric unit as a two-part disordered unit wherein the acidic hydrogen atom is bound to each ring. The two imidazolium cations share a strong hydrogen bond *via* the acidic hydrogen atom, which is disordered between two positions, being bonded to the first *versus* second imidazole ring in a 0.33 (2) to 0.67 (2) ratio. A benzene sulfonate anion is present for charge balance and inter­acts with the aromatic H atoms on both imidazole rings as well as with the methyl groups on the rings.

## Abstract 2

The title compound exists with one protonated imidazolium ring, one neutral imidazole ring, and a benzene­sulfonate anion in the asymmetric unit. The imidazole rings are held together through hydrogen bonding *via* a protonated nitro­gen on the ring.

## Graphical abstract

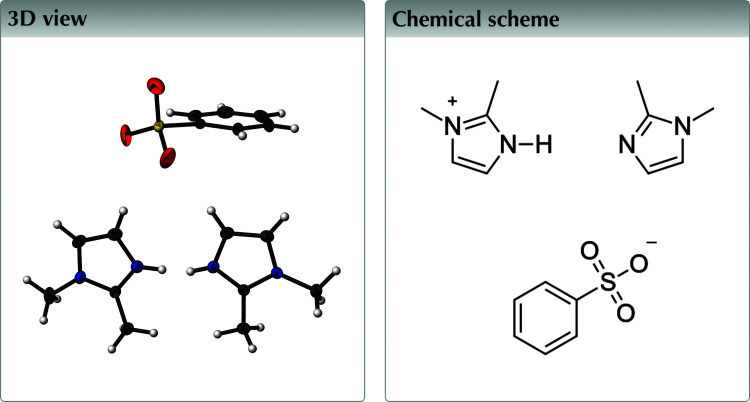

## Structure description

**Figure 1:**
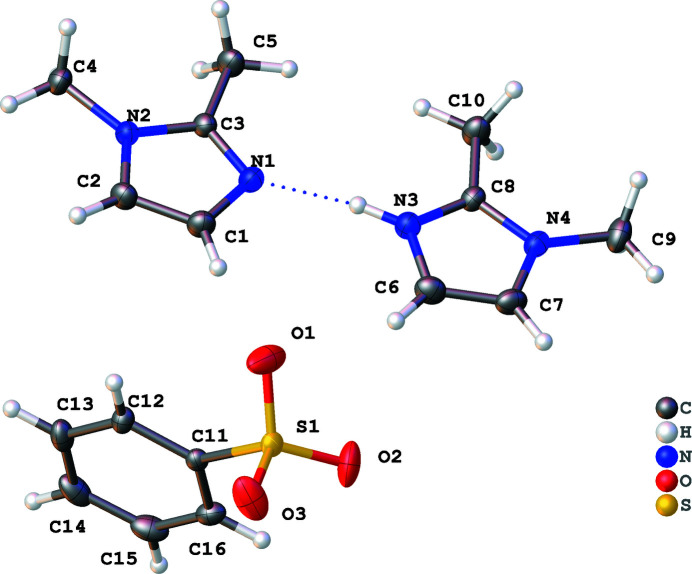
The title compound shown with 50% probability ellipsoids. Only the major component is shown.

**Figure 2:**
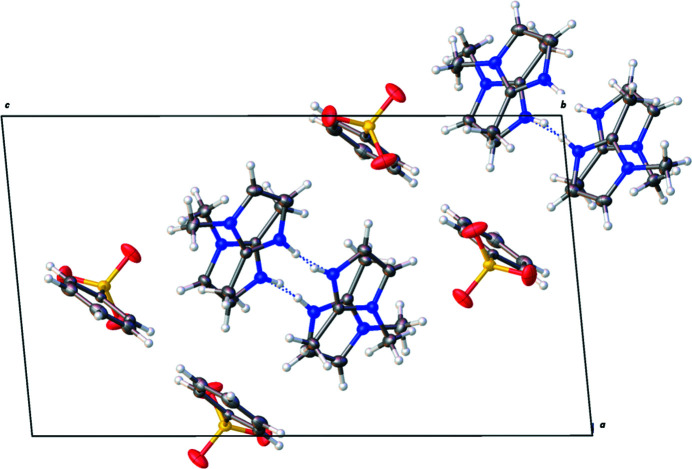
Packing diagram of the title compound viewed down the (010) plane showing a layered network of ion pairs held together through hydrogen inter­actions. Both parts of the disorder are shown.

The title compound (Fig. 1 ▸) crystallizes with two 1,2-di­methyl­imidazolium cations in the asymmetric unit. The two imidazole rings are each partially protonated, wherein the acidic hydrogen atom is bound between the two N atoms of the aromatic ring in a 0.33 (2) to 0.67 (2) ratio. Hydrogen bonding appears to the dominant inter­molecular inter­action with each molecule or ion exhibiting inter­actions (Fig. 2 ▸). For instance, the shortest hydrogen bonds are N—H⋯N links between the imidazolium rings with H⋯N = 1.83 (8) and 1.90 (8) Å. This bonding arises from the disordered hydrogen atom, which appears to be shared between the two rings. Further, cation–anion C—H⋯O inter­actions occur between the aromatic H atoms and the sulfonate O atoms. Finally, there are anion–anion inter­actions wherein O atoms of the sulfonate group interact with hydrogens on the benzene rings. A summary of the distances for the hydrogen bonds is found in Table 1 ▸.

For a related structure with a chloride anion, see Kelley *et al.* (2013 ▸).

## Synthesis and crystallization

The reaction was conducted in a Biotage Initiator+ microwave reactor. To a microwave vial was added a stir bar as well as 7.5 mmol (721 mg) of 1,2 di­methyl­imidazole and 7.5 mmol (901 μL) of benzene­sulfonyl fluoride. The vial was sealed and placed into the microwave reactor. The reaction was performed at 105°C for 5 minutes and 38 s with very high microwave absorption, stirring at 600 rpm. Once finished and cooled to room temperature, the solution was transferred to an oven-dried amber scintillation vial and sealed with parafilm. After one week, crystals suitable for diffraction were found growing in the vial.

**Figure 3:**
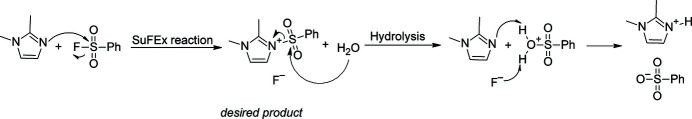
Proposed mechanism leading to the formation of the crystallized product reported herein.

A proposed mechanism leading to the formation of the crystallized product reported herein is shown in Fig. 3 ▸.

^1^H NMR (400 MHz, chloro­form-D) δ 8.01–7.99 (*m*, 1H), 7.89–7.87 (*m*, 1H), 7.77 (*dd*, *J* = 8.1, 6.7 Hz, 1H), 7.62 (*t*, *J* = 7.4 Hz, 1H), 7.35 (*t*, *J* = 2.6 Hz, 1H), 7.25 (*s*, 1H), 6.98–6.83 (*m*, 2H), 3.61 (*d*, *J* = 9.1 Hz, 3H), 2.46 (*d*, *J* = 14.0 Hz, 3H)

^13^C NMR (101 MHz, chloro­form-D) δ 206.7, 144.8, 135.7, 130.0, 129.8, 128.5, 128.3, 126.0, 121.2, 121.0, 77.4, 77.1, 76.8, 76.6, 33.5, 12.0, −1.6.

## Refinement

Crystal data, data collection and structure refinement details are summarized in Table 2 ▸.

## Supplementary Material

Crystal structure: contains datablock(s) I, global. DOI: 10.1107/S2414314620006896/bv4031sup1.cif

Structure factors: contains datablock(s) I. DOI: 10.1107/S2414314620006896/bv4031Isup2.hkl

Supporting information file. DOI: 10.1107/S2414314620006896/bv4031Isup3.cml

CCDC reference: 2005097

Additional supporting information:  crystallographic information; 3D view; checkCIF report

## Figures and Tables

**Table 1:** Hydrogen-bond geometry (Å, °)

*D*—H⋯*A*	*D*—H	H⋯*A*	*D*⋯*A*	*D*—H⋯*A*
N1—H1*N*⋯N3	0.83	1.90	2.6970 (11)	163
N3—H3*N*⋯N1	0.89	1.81	2.6970 (11)	170
C1—H1⋯O1	0.95	2.43	3.3741 (13)	170
C4—H4*B*⋯O3^i^	0.98	2.33	3.2956 (12)	170
C6—H6⋯O2	0.95	2.60	3.4288 (14)	146
C7—H7⋯O2^ii^	0.95	2.41	3.3254 (12)	162
C9—H9*A*⋯O3^iii^	0.98	2.53	3.4846 (14)	164
C9—H9*B*⋯O3^ii^	0.98	2.46	3.4381 (14)	173
C13—H13⋯O1^i^	0.95	2.67	3.4738 (14)	143
C14—H14⋯O1^iv^	0.95	2.48	3.3920 (13)	162

Symmetry codes: (i) 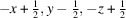 ; (ii) 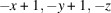 ; (iii) 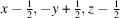 ; (iv) 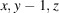 .

**Table 2:** Experimental details

Crystal data	
Chemical formula	C_5_H_9_N_2_^+^·C_6_H_5_O_3_S^−^·C_5_H_8_N_2_
*M*_r_	350.43
Crystal system, space group	Monoclinic, *P*2_1_/*n*
Temperature (K)	150
*a*, *b*, *c* (Å)	10.8820 (6), 8.4029 (4), 18.9678 (11)
β (°)	95.440 (2)
*V* (Å^3^)	1726.61 (16)
*Z*	4
Radiation type	Mo *K*α
μ (mm^−1^)	0.21
Crystal size (mm)	0.55 × 0.42 × 0.33

Data collection	
Diffractometer	Bruker AXS D8 Quest CMOS diffractometer
Absorption correction	Multi-scan (*SADABS*; Krause *et al.*, 2015 ▸)
*T*_min_, *T*_max_	0.716, 0.747
No. of measured, independent and observed [*I* > 2σ(*I*)] reflections	74762, 6612, 5787
*R*_int_	0.032
(sin θ/λ)_max_ (Å^−1^)	0.771

Refinement	
*R*[*F* ^2^ > 2σ(*F* ^2^)], *wR*(*F* ^2^), *S*	0.035, 0.095, 1.05
No. of reflections	6612
No. of parameters	225
H-atom treatment	H atoms treated by a mixture of independent and constrained refinement
Δρ_max_, Δρ_min_ (e Å^−3^)	0.40, −0.44

Computer programs: *APEX3* and *SAINT* (Bruker, 2018 ▸), *SHELXS97* (Sheldrick, 2008 ▸), *SHELXL2018/3* (Sheldrick, 2015 ▸), *shelXle* (Hübschle *et al.*, 2011 ▸), *OLEX2* (Dolomanov *et al.*, 2009 ▸), *publCIF* (Westrip, 2010 ▸) and *enCIFer* (Allen *et al.*, 2004 ▸).

## full crystallographic data

### Crystal data

**Table:**

C_5_H_9_N_2_^+^·C_6_H_5_O_3_S^−^·C_5_H_8_N_2_	*F*(000) = 744
*M**_r_* = 350.43	*D*_x_ = 1.348 Mg m^−^^3^
Monoclinic, *P*2_1_/*n*	Mo *K*α radiation, λ = 0.71073 Å
*a* = 10.8820 (6) Å	Cell parameters from 9689 reflections
*b* = 8.4029 (4) Å	θ = 2.7–33.2°
*c* = 18.9678 (11) Å	µ = 0.21 mm^−^^1^
β = 95.440 (2)°	*T* = 150 K
*V* = 1726.61 (16) Å^3^	Block, colourless
*Z* = 4	0.55 × 0.42 × 0.33 mm

### Data collection

**Table:**

Bruker AXS D8 Quest CMOS diffractometer	6612 independent reflections
Radiation source: fine focus sealed tube X-ray source	5787 reflections with *I* > 2σ(*I*)
Triumph curved graphite crystal monochromator	*R*_int_ = 0.032
Detector resolution: 10.4167 pixels mm^-1^	θ_max_ = 33.2°, θ_min_ = 2.3°
ω and phi scans	*h* = −16→16
Absorption correction: multi-scan (SADABS; Krause *et al.*, 2015)	*k* = −12→12
*T*_min_ = 0.716, *T*_max_ = 0.747	*l* = −29→28
74762 measured reflections	

### Refinement

**Table:**

Refinement on *F*^2^	Secondary atom site location: difference Fourier map
Least-squares matrix: full	Hydrogen site location: inferred from neighbouring sites
*R*[*F*^2^ > 2σ(*F*^2^)] = 0.035	H atoms treated by a mixture of independent and constrained refinement
*wR*(*F*^2^) = 0.095	*w* = 1/[σ^2^(*F*_o_^2^) + (0.0417*P*)^2^ + 0.6618*P*] where *P* = (*F*_o_^2^ + 2*F*_c_^2^)/3
*S* = 1.05	(Δ/σ)_max_ = 0.001
6612 reflections	Δρ_max_ = 0.40 e Å^−^^3^
225 parameters	Δρ_min_ = −0.44 e Å^−^^3^
0 restraints	Extinction correction: SHELXL-2018/3 (Sheldrick 2015), Fc^*^=kFc[1+0.001xFc^2^λ^3^/sin(2θ)]^-1/4^
Primary atom site location: structure-invariant direct methods	Extinction coefficient: 0.0085 (10)

### Special details

**Table:**

Geometry. All esds (except the esd in the dihedral angle between two l.s. planes) are estimated using the full covariance matrix. The cell esds are taken into account individually in the estimation of esds in distances, angles and torsion angles; correlations between esds in cell parameters are only used when they are defined by crystal symmetry. An approximate (isotropic) treatment of cell esds is used for estimating esds involving l.s. planes.
Refinement. H atoms attached to carbon atoms were positioned geometrically and constrained to ride on their parent atoms. C—H bond distances were constrained to 0.95 Å for aromatic and alkene C—H moieties, and to 0.98 Å for CH_3_ moieties, respectively. The N—H proton hydrogen bonding between atoms N1 and N3 was found to be disordered and was refined as split between two positions. The H atoms were assigned as bonded to a planar (*sp*^2^ hybridized) N atom, respectively with fixed bond angles and torsion angles, but the N—H bond distances were allowed to refine to account for asymmetry induced by charge and hydrogen bonding (AFIX 44 command). N—H distances refined to 0.83 (5) for N1—H1 and to 0.89 (2) for N3—H3, occupancies refined to 0.33 (2) for H1 and 0.67 (2) for H3. Methyl CH_3_ were allowed to rotate but not to tip to best fit the experimental electron density. *U*_iso_(H) values were set to a multiple of *U*_eq_(C/N) with 1.5 for CH_3_ and 1.2 for C—H and NH^+^, respectively.

### Fractional atomic coordinates and isotropic or equivalent isotropic displacement parameters (Å^2^)

**Table:**

	*x*	*y*	*z*	*U*_iso_*/*U*_eq_	Occ. (<1)
C1	0.07995 (8)	0.11974 (12)	0.12505 (5)	0.02370 (17)	
H1	0.162507	0.154570	0.136425	0.028*	
C2	0.01650 (8)	0.02111 (12)	0.16550 (5)	0.02268 (16)	
H2	0.045537	−0.025178	0.209661	0.027*	
C3	−0.10216 (8)	0.08832 (10)	0.06876 (4)	0.01832 (14)	
C4	−0.20228 (9)	−0.08561 (12)	0.15402 (5)	0.02410 (17)	
H4A	−0.264761	−0.010298	0.167446	0.036*	
H4B	−0.172925	−0.150361	0.195187	0.036*	
H4C	−0.238614	−0.154975	0.116071	0.036*	
C5	−0.21283 (8)	0.09673 (13)	0.01670 (5)	0.02515 (18)	
H5A	−0.194381	0.162346	−0.023687	0.038*	
H5B	−0.281506	0.144135	0.039231	0.038*	
H5C	−0.235631	−0.010751	0.000206	0.038*	
C6	0.23459 (9)	0.40162 (12)	−0.00734 (5)	0.02566 (18)	
H6	0.285770	0.372447	0.034004	0.031*	
C7	0.26716 (8)	0.49589 (12)	−0.06067 (6)	0.02502 (18)	
H7	0.345007	0.545291	−0.063695	0.030*	
C8	0.07312 (8)	0.42021 (10)	−0.08640 (4)	0.01863 (14)	
C9	0.15832 (10)	0.59191 (13)	−0.17707 (5)	0.02847 (19)	
H9A	0.142896	0.516294	−0.216246	0.043*	
H9B	0.236534	0.647282	−0.181302	0.043*	
H9C	0.090972	0.669653	−0.178780	0.043*	
C10	−0.05084 (9)	0.40053 (13)	−0.12482 (5)	0.02596 (18)	
H10A	−0.090249	0.504931	−0.131824	0.039*	
H10B	−0.101453	0.332570	−0.097120	0.039*	
H10C	−0.042876	0.351054	−0.170954	0.039*	
C11	0.43346 (7)	0.00325 (9)	0.14618 (4)	0.01514 (13)	
C12	0.36079 (8)	−0.06738 (10)	0.19405 (4)	0.01852 (14)	
H12	0.326061	−0.004339	0.228696	0.022*	
C13	0.33919 (9)	−0.23035 (11)	0.19101 (5)	0.02475 (18)	
H13	0.289972	−0.278763	0.223810	0.030*	
C14	0.38933 (10)	−0.32262 (12)	0.14020 (6)	0.0293 (2)	
H14	0.375570	−0.434269	0.138722	0.035*	
C15	0.45951 (9)	−0.25157 (13)	0.09161 (6)	0.02817 (19)	
H15	0.492304	−0.314441	0.056220	0.034*	
C16	0.48217 (8)	−0.08834 (11)	0.09445 (5)	0.02151 (16)	
H16	0.530578	−0.039928	0.061247	0.026*	
N1	0.00522 (7)	0.16129 (10)	0.06471 (4)	0.02100 (14)	
H1N	0.0238 (11)	0.220 (3)	0.0325 (19)	0.025*	0.33 (2)
N2	−0.09869 (7)	0.00209 (9)	0.12923 (4)	0.01850 (13)	
N3	0.11314 (7)	0.35581 (10)	−0.02425 (4)	0.02125 (14)	
H3N	0.0690 (11)	0.2936 (16)	0.0021 (7)	0.026*	0.67 (2)
N4	0.16511 (7)	0.50600 (9)	−0.10967 (4)	0.02018 (14)	
O1	0.35734 (8)	0.28443 (9)	0.17511 (6)	0.0439 (2)	
O2	0.49875 (9)	0.26229 (11)	0.08516 (4)	0.0383 (2)	
O3	0.57130 (9)	0.21999 (10)	0.20780 (5)	0.0400 (2)	
S1	0.46799 (2)	0.20953 (2)	0.15392 (2)	0.01791 (6)	

### Atomic displacement parameters (Å^2^)

**Table:**

	*U*^11^	*U*^22^	*U*^33^	*U*^12^	*U*^13^	*U*^23^
C1	0.0187 (4)	0.0277 (4)	0.0242 (4)	−0.0014 (3)	−0.0009 (3)	0.0018 (3)
C2	0.0207 (4)	0.0263 (4)	0.0202 (4)	0.0008 (3)	−0.0024 (3)	0.0030 (3)
C3	0.0179 (3)	0.0208 (4)	0.0163 (3)	0.0012 (3)	0.0019 (3)	0.0019 (3)
C4	0.0218 (4)	0.0268 (4)	0.0244 (4)	−0.0017 (3)	0.0057 (3)	0.0059 (3)
C5	0.0206 (4)	0.0326 (5)	0.0214 (4)	−0.0007 (3)	−0.0025 (3)	0.0057 (3)
C6	0.0217 (4)	0.0257 (4)	0.0285 (4)	−0.0007 (3)	−0.0032 (3)	−0.0017 (3)
C7	0.0176 (4)	0.0236 (4)	0.0338 (5)	−0.0029 (3)	0.0020 (3)	−0.0033 (3)
C8	0.0182 (3)	0.0197 (3)	0.0183 (3)	−0.0019 (3)	0.0032 (3)	−0.0005 (3)
C9	0.0367 (5)	0.0263 (4)	0.0240 (4)	−0.0061 (4)	0.0108 (4)	0.0020 (3)
C10	0.0209 (4)	0.0326 (5)	0.0237 (4)	−0.0051 (3)	−0.0017 (3)	0.0037 (3)
C11	0.0138 (3)	0.0151 (3)	0.0160 (3)	−0.0013 (2)	−0.0009 (2)	0.0004 (2)
C12	0.0195 (3)	0.0185 (3)	0.0174 (3)	−0.0033 (3)	0.0012 (3)	0.0012 (3)
C13	0.0264 (4)	0.0195 (4)	0.0271 (4)	−0.0066 (3)	−0.0035 (3)	0.0064 (3)
C14	0.0310 (5)	0.0154 (4)	0.0388 (5)	0.0007 (3)	−0.0104 (4)	−0.0017 (3)
C15	0.0254 (4)	0.0259 (4)	0.0320 (5)	0.0068 (3)	−0.0036 (3)	−0.0108 (4)
C16	0.0171 (3)	0.0271 (4)	0.0203 (4)	0.0017 (3)	0.0017 (3)	−0.0031 (3)
N1	0.0185 (3)	0.0238 (3)	0.0208 (3)	−0.0010 (3)	0.0027 (2)	0.0026 (3)
N2	0.0179 (3)	0.0203 (3)	0.0174 (3)	0.0002 (2)	0.0017 (2)	0.0026 (2)
N3	0.0206 (3)	0.0230 (3)	0.0199 (3)	−0.0013 (3)	0.0008 (2)	0.0010 (3)
N4	0.0199 (3)	0.0193 (3)	0.0219 (3)	−0.0027 (2)	0.0051 (2)	−0.0011 (3)
O1	0.0321 (4)	0.0167 (3)	0.0868 (7)	−0.0008 (3)	0.0267 (5)	−0.0041 (4)
O2	0.0559 (5)	0.0311 (4)	0.0288 (4)	−0.0159 (4)	0.0089 (4)	0.0088 (3)
O3	0.0445 (5)	0.0265 (4)	0.0438 (5)	−0.0125 (3)	−0.0235 (4)	0.0020 (3)
S1	0.01652 (9)	0.01532 (9)	0.02171 (10)	−0.00380 (6)	0.00082 (7)	0.00194 (6)

### Geometric parameters (Å, º)

**Table:**

C1—C2	1.3612 (13)	C9—H9A	0.9800
C1—N1	1.3845 (12)	C9—H9B	0.9800
C1—H1	0.9500	C9—H9C	0.9800
C2—N2	1.3809 (11)	C10—H10A	0.9800
C2—H2	0.9500	C10—H10B	0.9800
C3—N1	1.3283 (11)	C10—H10C	0.9800
C3—N2	1.3541 (11)	C11—C16	1.3910 (12)
C3—C5	1.4848 (12)	C11—C12	1.3921 (11)
C4—N2	1.4613 (11)	C11—S1	1.7767 (8)
C4—H4A	0.9800	C12—C13	1.3897 (12)
C4—H4B	0.9800	C12—H12	0.9500
C4—H4C	0.9800	C13—C14	1.3883 (15)
C5—H5A	0.9800	C13—H13	0.9500
C5—H5B	0.9800	C14—C15	1.3866 (16)
C5—H5C	0.9800	C14—H14	0.9500
C6—C7	1.3580 (14)	C15—C16	1.3937 (14)
C6—N3	1.3847 (12)	C15—H15	0.9500
C6—H6	0.9500	C16—H16	0.9500
C7—N4	1.3815 (12)	N1—H1N	0.83 (5)
C7—H7	0.9500	N3—H3N	0.89 (2)
C8—N3	1.3322 (11)	O1—S1	1.4489 (8)
C8—N4	1.3418 (11)	O2—S1	1.4465 (8)
C8—C10	1.4805 (12)	O3—S1	1.4484 (8)
C9—N4	1.4639 (12)		
			
C2—C1—N1	109.27 (8)	C8—C10—H10C	109.5
C2—C1—H1	125.4	H10A—C10—H10C	109.5
N1—C1—H1	125.4	H10B—C10—H10C	109.5
C1—C2—N2	105.94 (8)	C16—C11—C12	120.15 (8)
C1—C2—H2	127.0	C16—C11—S1	120.42 (6)
N2—C2—H2	127.0	C12—C11—S1	119.39 (6)
N1—C3—N2	110.00 (7)	C13—C12—C11	119.83 (8)
N1—C3—C5	127.01 (8)	C13—C12—H12	120.1
N2—C3—C5	122.99 (8)	C11—C12—H12	120.1
N2—C4—H4A	109.5	C14—C13—C12	120.20 (9)
N2—C4—H4B	109.5	C14—C13—H13	119.9
H4A—C4—H4B	109.5	C12—C13—H13	119.9
N2—C4—H4C	109.5	C15—C14—C13	119.89 (9)
H4A—C4—H4C	109.5	C15—C14—H14	120.1
H4B—C4—H4C	109.5	C13—C14—H14	120.1
C3—C5—H5A	109.5	C14—C15—C16	120.31 (9)
C3—C5—H5B	109.5	C14—C15—H15	119.8
H5A—C5—H5B	109.5	C16—C15—H15	119.8
C3—C5—H5C	109.5	C11—C16—C15	119.60 (9)
H5A—C5—H5C	109.5	C11—C16—H16	120.2
H5B—C5—H5C	109.5	C15—C16—H16	120.2
C7—C6—N3	107.48 (8)	C3—N1—C1	106.66 (8)
C7—C6—H6	126.3	C3—N1—H1N	126.7
N3—C6—H6	126.3	C1—N1—H1N	126.7
C6—C7—N4	106.69 (8)	C3—N2—C2	108.13 (7)
C6—C7—H7	126.7	C3—N2—C4	125.59 (7)
N4—C7—H7	126.7	C2—N2—C4	126.12 (7)
N3—C8—N4	108.53 (8)	C8—N3—C6	108.47 (8)
N3—C8—C10	126.61 (8)	C8—N3—H3N	125.8
N4—C8—C10	124.85 (8)	C6—N3—H3N	125.8
N4—C9—H9A	109.5	C8—N4—C7	108.83 (8)
N4—C9—H9B	109.5	C8—N4—C9	125.08 (8)
H9A—C9—H9B	109.5	C7—N4—C9	126.06 (8)
N4—C9—H9C	109.5	O2—S1—O3	112.77 (6)
H9A—C9—H9C	109.5	O2—S1—O1	112.73 (6)
H9B—C9—H9C	109.5	O3—S1—O1	112.82 (7)
C8—C10—H10A	109.5	O2—S1—C11	106.84 (4)
C8—C10—H10B	109.5	O3—S1—C11	105.15 (4)
H10A—C10—H10B	109.5	O1—S1—C11	105.78 (4)
			
N1—C1—C2—N2	0.06 (11)	C1—C2—N2—C3	−0.06 (10)
N3—C6—C7—N4	−0.20 (11)	C1—C2—N2—C4	−175.75 (9)
C16—C11—C12—C13	1.40 (12)	N4—C8—N3—C6	−0.12 (10)
S1—C11—C12—C13	−176.27 (7)	C10—C8—N3—C6	179.13 (9)
C11—C12—C13—C14	−0.33 (13)	C7—C6—N3—C8	0.20 (11)
C12—C13—C14—C15	−1.04 (14)	N3—C8—N4—C7	0.00 (10)
C13—C14—C15—C16	1.35 (15)	C10—C8—N4—C7	−179.28 (9)
C12—C11—C16—C15	−1.09 (12)	N3—C8—N4—C9	178.37 (8)
S1—C11—C16—C15	176.55 (7)	C10—C8—N4—C9	−0.91 (14)
C14—C15—C16—C11	−0.28 (14)	C6—C7—N4—C8	0.13 (10)
N2—C3—N1—C1	0.00 (10)	C6—C7—N4—C9	−178.22 (9)
C5—C3—N1—C1	179.55 (9)	C16—C11—S1—O2	24.77 (8)
C2—C1—N1—C3	−0.04 (11)	C12—C11—S1—O2	−157.57 (7)
N1—C3—N2—C2	0.03 (10)	C16—C11—S1—O3	−95.30 (8)
C5—C3—N2—C2	−179.54 (9)	C12—C11—S1—O3	82.36 (8)
N1—C3—N2—C4	175.76 (8)	C16—C11—S1—O1	145.10 (8)
C5—C3—N2—C4	−3.81 (14)	C12—C11—S1—O1	−37.24 (9)

### Hydrogen-bond geometry (Å, º)

**Table:**

*D*—H···*A*	*D*—H	H···*A*	*D*···*A*	*D*—H···*A*
N1—H1*N*···N3	0.83	1.90	2.6970 (11)	163
N3—H3*N*···N1	0.89	1.81	2.6970 (11)	170
C1—H1···O1	0.95	2.43	3.3741 (13)	170
C4—H4*B*···O3^i^	0.98	2.33	3.2956 (12)	170
C6—H6···O2	0.95	2.60	3.4288 (14)	146
C7—H7···O2^ii^	0.95	2.41	3.3254 (12)	162
C9—H9*A*···O3^iii^	0.98	2.53	3.4846 (14)	164
C9—H9*B*···O3^ii^	0.98	2.46	3.4381 (14)	173
C13—H13···O1^i^	0.95	2.67	3.4738 (14)	143
C14—H14···O1^iv^	0.95	2.48	3.3920 (13)	162

Symmetry codes: (i) −*x*+1/2, *y*−1/2, −*z*+1/2; (ii) −*x*+1, −*y*+1, −*z*; (iii) *x*−1/2, −*y*+1/2, *z*−1/2; (iv) *x*, *y*−1, *z*.
